# QiShenYiQi pill attenuates atherosclerosis by promoting regulatory T cells, inhibiting T helper 17 cells and accelerating cholesterol excretion

**DOI:** 10.18632/oncotarget.19072

**Published:** 2017-07-07

**Authors:** Li Peng, Chong-Shan Lv, Yun Zhao, Shao-Dong Chen, Yang Huang, Da-Wei Lu, Shu-Qiong Huang, Zong-Bao Yang, Lin-Chao Qian, Lei Wen

**Affiliations:** ^1^ Department of Traditional Chinese Medicine, Medical College, Xiamen University, Xiamen 361102, China

**Keywords:** atherosclerosis, regulatory T cell, interleukin-17, cholesterol excretion, QiShenYiQi

## Abstract

**Objective:**

The aim of this study was to explore potential immunoregulatory mechanisms underlying the suppressive effect on atherosclerosis of QiShenYiQi pill (QSYQ).

**Methods and Results:**

Male ApoE^-/-^ mice were maintained on a Western-type diet and QSYQ treatment for eight weeks. Determination of atherosclerosis demonstrated that QSYQ attenuated plaque formation and decreased the level of blood low-density lipoproteins-cholesterol. QSYQ treatment did not affect body weight but reduced the ratio of liver weight and body weight. Western blots of liver showed that QSYQ increased the expression of liver X receptor alpha and ATP-binding cassette sub-family G member 5. Western blots of atherosclerotic aorta revealed that QSYQ inhibited the expression of cluster of differentiation 36, promoted the expression of forkhead box P3 and decreased interleukin-17A expression. Western blots of spleen showed that QSYQ decreased the expression of mothers against decapentaplegic homolog 2/3 and forkhead box P3, as well as attenuated the expression of spleen interleukin-6, RAR-related orphan receptor gamma and interleukin-17A.

**Conclusions:**

QSYQ exerted an anti-atherosclerosis effect by promoting regulatory T cells in atherosclerotic lesion, inhibiting T helper 17 cells in plaque and spleen and accelerating liver cholesterol excretion.

## INTRODUCTION

Atherosclerosis is a chronic inflammatory disease [1]. Immune responses participate in every phase of atherosclerosis [2]. Published evidence shows that both innate and adaptive immune responses are involved in atherogenesis, but the precise molecular mechanisms of these responses are not well understood [3]. When low-density lipoproteins (LDL) accumulate in the intima, endothelial cells become activated and express cell adhesion molecules, chemokines and other mediators, which promote circulating monocyte rolling on and adhering to the endothelium. Subsequently, monocytes infiltrate the subendothelial space to initiate the formation of atherosclerotic plaques. Mononuclear macrophages can take up oxidized-LDL through scavenger receptors, such as cluster of differentiation 36 (CD36) [4, 5], and/or release toll-like receptor-mediated pro-inflammatory cytokines, chemokines, and proteases during innate immune responses [6].

Naïve CD4^+^ T cells migrate into the diseased intima and develop into effector T cells [7, 8]. Natural regulatory T cells (Treg) play a protective role in patients with coronary heart disease and in ApoE^-/-^ mice fed with high cholesterol diet [9, 10]. The forkhead box P3 (Foxp3) has been identified to regulate the development and function of Treg cells. Foxp3^+^ Treg cells can be activated by transforming growth factor beta 1 (TGF-β1) to produce TGF-β1. In addition, TGF-β1 suppresses the function of T helper 1, 2, 17 cell (Th1, Th2, Th17) [11]. The immunosuppressive effect of Foxp3^+^ Treg cells inhibit monocyte adherence to the surface of arterial endothelial cells and thereby block their infiltration into the subendothelial space, resulting in the reduction of atherosclerotic lesion formation [10]. Depletion of Foxp3^+^ Treg cells promotes hypercholesterolemia and atherosclerosis [12]. Interleukin-17 (IL-17) is the major effector cytokine secreted by Th17 cells, which play a pro-atherogenic role in ApoE^-/-^ mice by inducing aortic chemokines and accelerating neutrophil and monocyte recruitment into the arterial wall [13]. In addition, elevated blood cholesterol can be transported to the liver and taken up into hepatocytes via the LDL receptor (LDLR), and then it is excreted into bile by mechanisms that depend on liver X receptor alpha (LXR-α) and ATP-binding cassette sub-family G member 5 (ABCG5) [14].

QiShenYiQi pill (QSYQ) is used in Chinese medicine approved by the China food and drug administration in 2003 for the treatment of cardiovascular diseases [15]. The mechanism of QSYQ was believed to involve attenuation of the AngII-NADPH oxidase pathway and inhibition of inflammatory pathways via energy modulation [16, 17]. The major ingredients of QSYQ include astragaloside IV, salvianolic acid B, Notoginsenoside R1, etc. Astragaloside IV inhibits vascular smooth muscle cell proliferation and migration *in vitro*, and decreases high mobility group box 1 protein-induced inflammation by promoting Foxp3^+^ Treg *in vivo* [18, 19]. However, the exact immunoregulatory mechanism underlying the effect of QSYQ largely remains to be identified. In this study, we investigated mechanisms of QSYQ on the immune system and distinct cytokines involved in atherosclerosis using ApoE^-/-^ mice.

## RESULTS

### QSYQ attenuated atherosclerotic lesion formation in ApoE^-/-^ mice

To evaluate the effect of QSYQ on the development of atherosclerosis, we treated 8w male ApoE^-/-^ mice with a low dose (human equivalent) or high dose (five times of human equivalent) QSYQ for 8 weeks. During the experiments, no adverse effect was observed.

**Figure 1:**
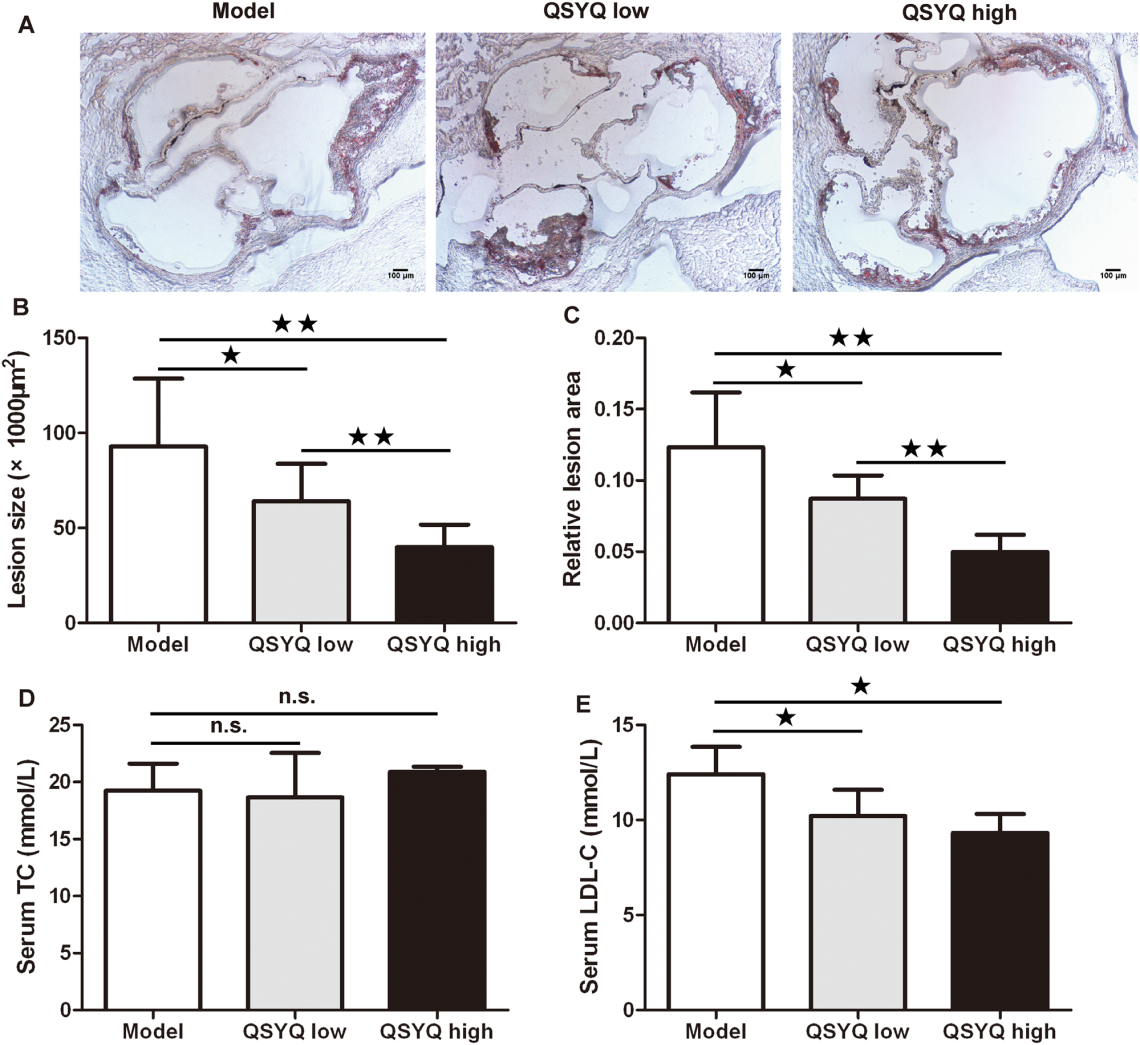
Effect of QSYQ on atherosclerotic lesion and blood lipids in ApoE^-/-^ mice **(A)** Representative photomicrographs of Sudan IV staining in aortic root of male ApoE^-/-^ mice treated with QSYQ or control saline. **(B** and **C)** The quantitative comparison of atherosclerotic lesion size and relative lesion area between drug treatment groups and model group (n=6). **(D** and **E)** The serum TC and LDL-C of ApoE^-/-^ mice were detected and compared between drug treatment groups and model group (n=6). Data were showed as mean ± standard deviation and compared by one-way analysis of variance and following with Fisher’s Least Significant Difference test for individual comparisons. ^n.s.^p > 0.05; ^★^p < 0.05; ^★★^p < 0.01.

Aortic root was examined for atherosclerosis. Frozen sections of aortic root were stained with Sudan IV and analyzed quantitatively. The aortic root of ApoE^-/-^ mice showed lipid-rich atherosclerotic plaques (Figure 1A). QSYQ showed at most 56% reduction in the size of atherosclerotic lesions in aortic root (40.38×10^3^±12.67×10^3^μm^2^ versus 92.80×10^3^±35.74×10^3^μm^2^) and 58% reduction in relative lesion area (0.05±0.01 versus 0.12±0.04) compared with saline-treated controls (*P* < 0.01) (Figure 1B and 1C).

We also investigated the effect of QSYQ on the level of blood lipids in atherosclerotic mice. We found no significant difference in serum total cholesterol (TC) between drug treatment groups and model group at the end of the experiment (*P* > 0.05). Interestingly, both doses of QSYQ showed significant reductions of serum LDL cholesterol (LDL-C) compared with model group (*P* < 0.05) (Figure 1D and 1E).

To further validate the anti-atherosclerosis effect of QSYQ, we next performed western blotting analyses in whole aorta to determine the protein expression of CD36. CD36 was a scavenger receptor on the surface of macrophage that binded oxidized LDL and contributed to the pathogenesis of atherosclerosis [20]. We found that QSYQ significant reduced the expression of CD36 in two dose groups compared with that in model group (*P* < 0.05) (Figure 3B).

**Figure 2:**
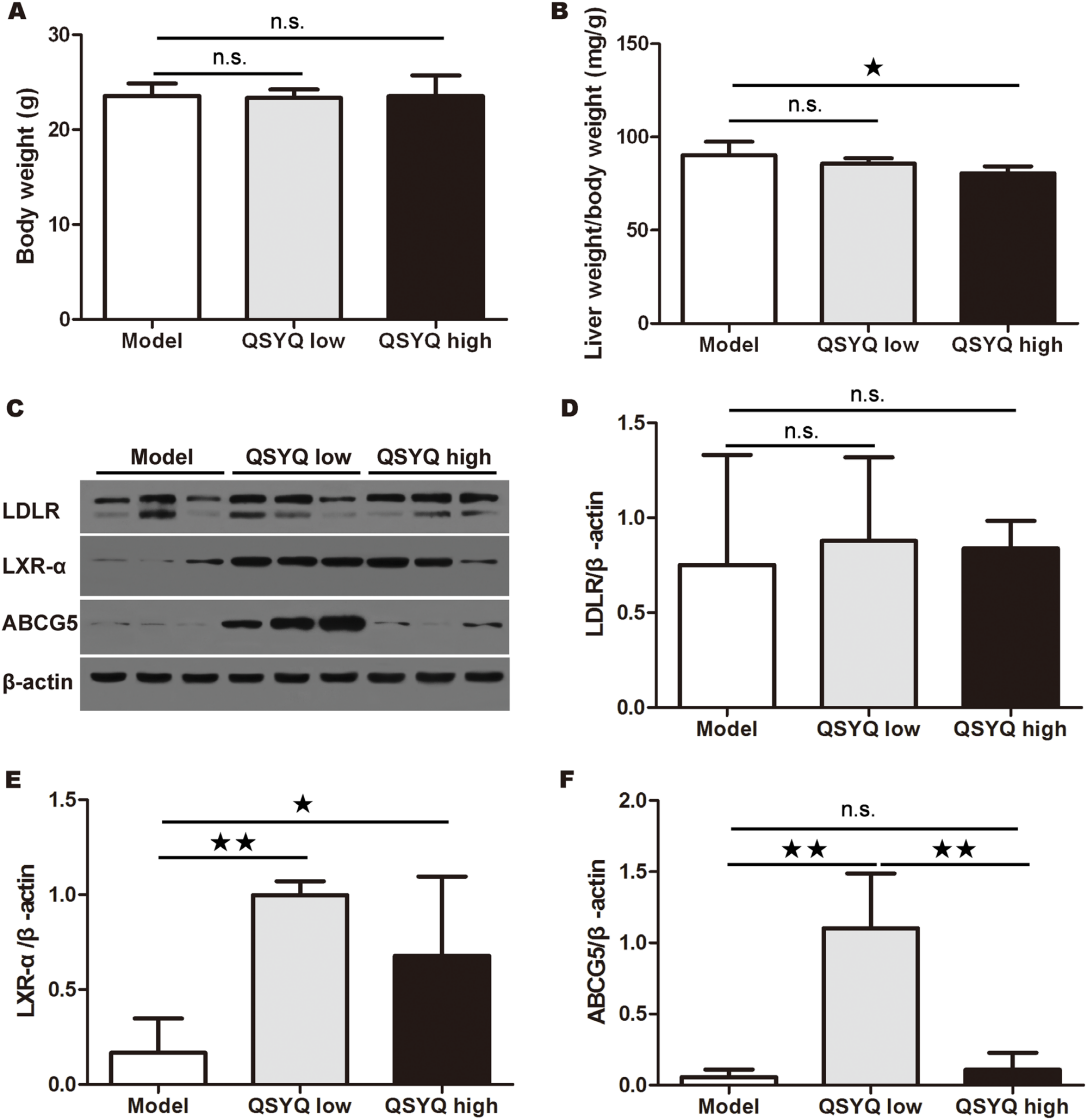
Effect of QSYQ on liver weight and liver cholesterol excretion pathway of ApoE^-/-^ mice **(A** and **B)** After eight weeks western-type diet and QSYQ treatment, the body weight and the ratio of liver weight versus body weight of ApoE^-/-^ mice were recorded and compared between drug treatment groups and model group (n=6). **(C)** The expression of LDLR, LXR-αand ABCG5 in liver of ApoE^-/-^ mice were detected by western blotting. **(D**, **E** and **F)** The quantitative comparison of LDLR, LXR-αand ABCG5 between drug treatment groups and model group (n=3). Data were showed as mean ± standard deviation and compared by one-way analysis of variance and following with Fisher’s Least Significant Difference test for individual comparisons. ^n.s.^p > 0.05; ^★^p < 0.05; ^★★^p < 0.01.

**Figure 3:**
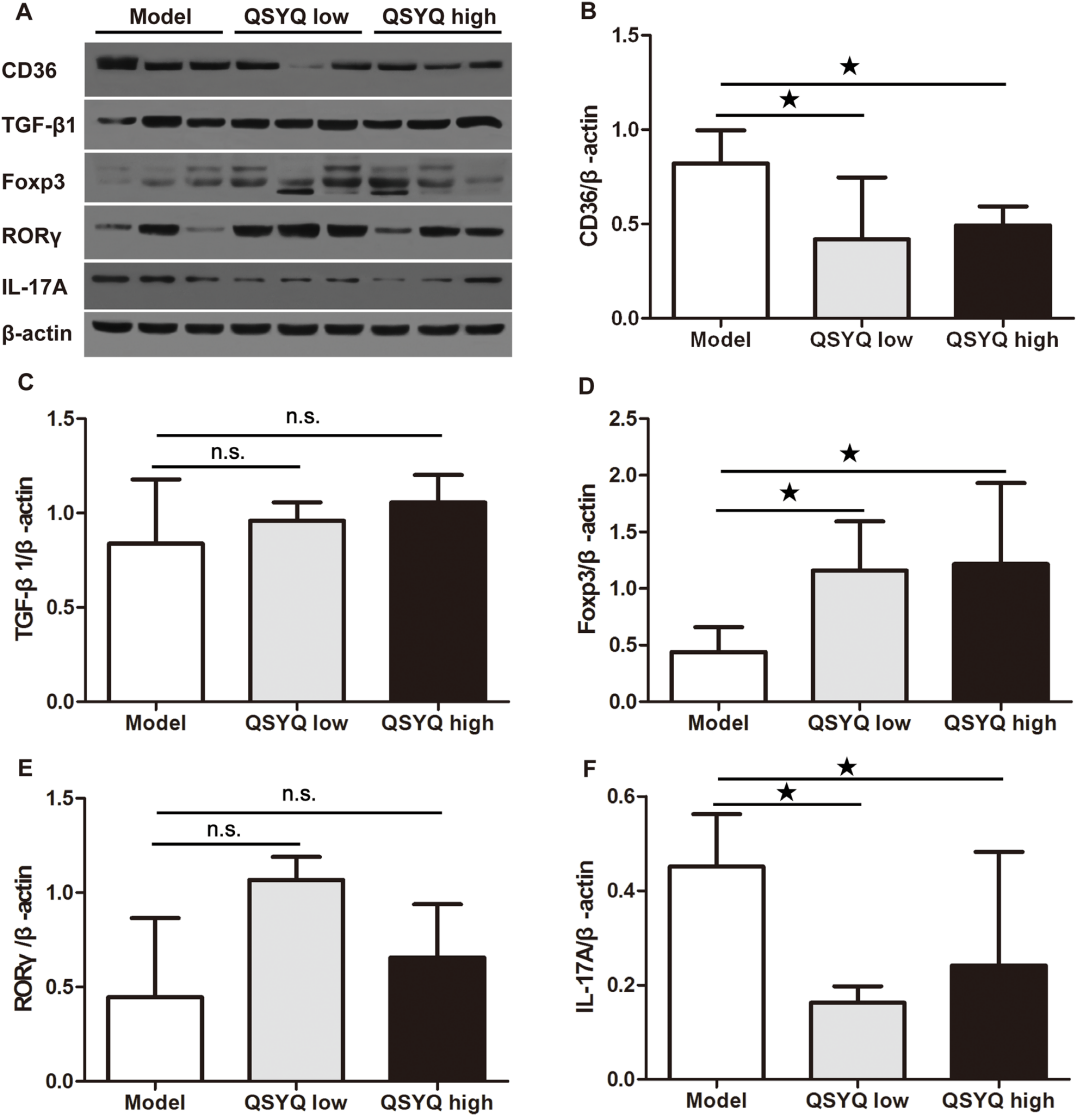
Effect of QSYQ on regulatory T cells and T helper 17 cells in atherosclerotic aorta of ApoE^-/-^ mice **(A)** The expression of CD36 (macrophage-related), TGF-β1/Foxp3 (regulatory T cells-related) and RORγ/IL-17A (T helper 17 cells-related) in atherosclerotic aorta of ApoE^-/-^ mice were detected by western blotting. **(B**, **C, D, E** and **F)** The quantitative comparison of CD36, TGF-β1, Foxp3, RORγ and IL-17A between drug treatment groups and model group (n=3). Data were showed as mean ± standard deviation and compared by one-way analysis of variance and following with Fisher’s Least Significant Difference test for individual comparisons. ^n.s.^p > 0.05; ^★^p < 0.05.

These results suggested that QSYQ reduced the development of atherosclerosis by inhibiting blood LDL-C and macrophage uptake of oxidized LDL.

### QSYQ promoted liver cholesterol excretion pathway

As ApoE gene knockout mice showed reduced HDL production and attenuated liver cholesterol excretion via the scavenger receptor class B member 1 pathway, an alternative pathway involved the LDLR-LXR-α-ABCG5 pathway, which had been shown to remove cholesterol from the blood and thereby reducing the risk of atherosclerosis in ApoE^-/-^ mice [14, 21].

To determine the potential mechanisms for QSYQ-dependent reduction of atherosclerotic lesion and serum LDL-C, we examined liver weight and in particular the liver cholesterol excretion pathway by western blotting. The result showed that there was no significant difference in mice body weight after eight weeks QSYQ treatment (*P* > 0.05). High dose of QSYQ decreased the ratio of liver weight versus body weight compared with model group (*P* < 0.05) (Figure 2A and 2B). We found no significant difference in the expression of LDLR in liver between drug treatment groups and model group (*P* > 0.05). Interestingly, two doses of QSYQ promoted the expression of LXR-α in liver compared with model group (*P* < 0.05 or 0.01). The low dose of QSYQ also increased the expression of ABCG5 in liver (*P* < 0.01) (Figure 2C-2F).

These results suggested that QSYQ treatment might remove blood cholesterol by promoting the LDLR-LXR-α-ABCG5 pathway in liver.

### QSYQ promoted treg cells and inhibited Th17 cells in atherosclerotic plaque

Foxp3 had been identified as the key marker for defining natural Treg cells, which could regulate Treg development and function [22]. It was reported that Foxp3^+^ Treg cells inhibited Th1/Th2/Th17 immune responses and played an important role in reducing atherosclerotic lesions [11]. In contrast, previous studies showed that IL-17, the major effector cytokine of Th17, was shown to be proatherogenic by promoting monocyte/macrophage recruitment into the arterial wall, which was inhibited by Treg cells [23]

To identify the potential mechanisms of QSYQ-dependent atherosclerosis development, we investigated the expression of Treg-regulating proteins in atherosclerotic aorta by Western blot. We found no significant difference in the TGF-β1 protein expression in QSYQ treated mice compared with ApoE^-/-^ mice (*P* > 0.05). However, QSYQ up-regulated the expression of Foxp3 compared to ApoE^-/-^ mice (*P* < 0.05) (Figure 3C and 3D).

We further examined Th17-regulating proteins in whole aorta extracts by Western blot. After eight weeks of drug treatment, we found no significant difference in the expression of RAR-related orphan receptor gamma (RORγ) in the aorta (*P* > 0.05). However, two doses of QSYQ significantly down-regulated the expression of IL-17A in the atherosclerotic aorta (*P* < 0.05) (Figure 3E and 3F).

These results suggested that QSYQ inhibited the development of atherosclerosis by promoting Treg cells and inhibiting Th17 cells in local lesion.

### QSYQ inhibited treg cells and Th17 cells in spleen

**Figure 4:**
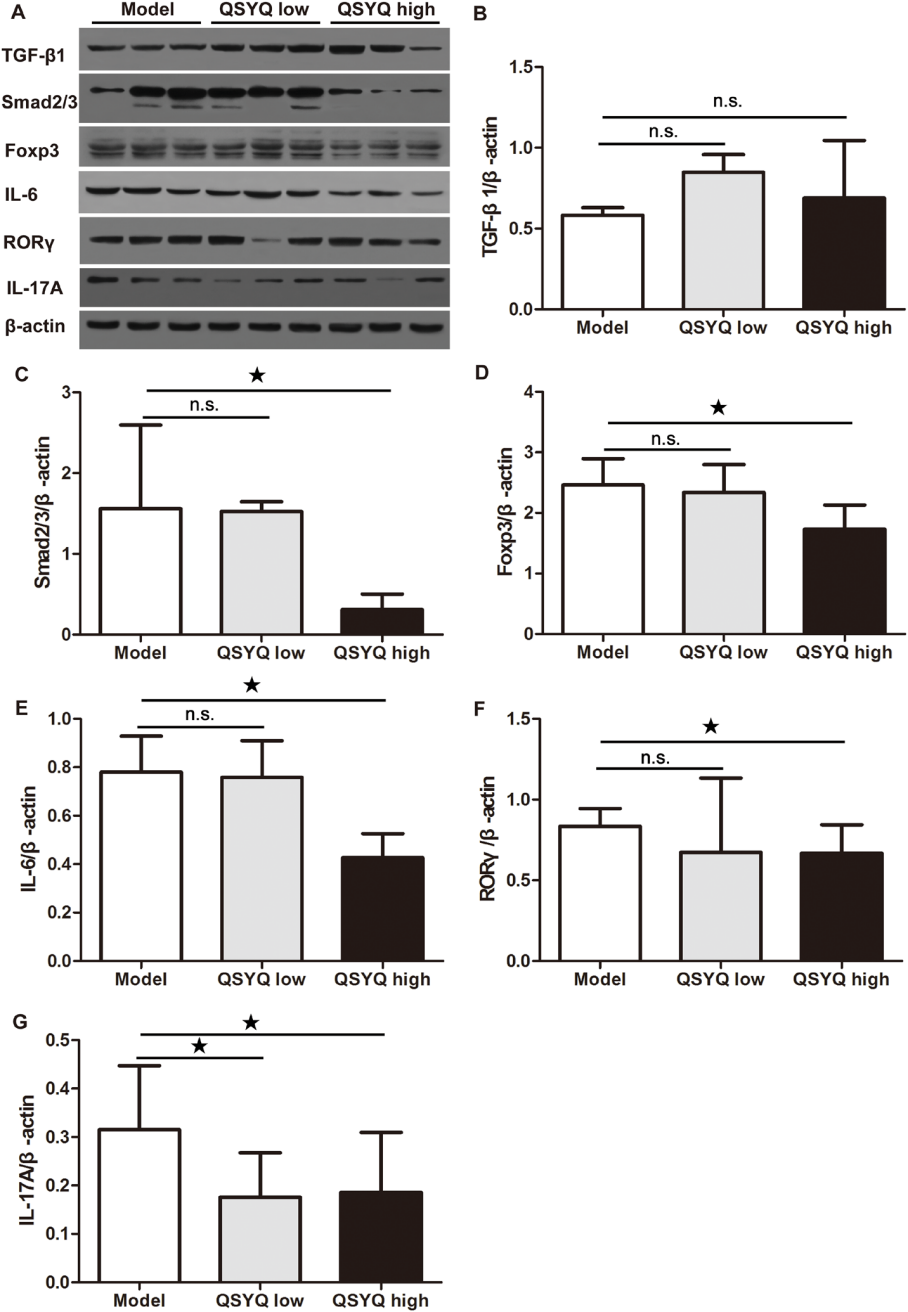
Effect of QSYQ on regulatory T cells and T helper 17 cells in spleen of ApoE^-/-^ mice **(A)** The expression of TGF-β1/Smad2/3/Foxp3 (regulatory T cells-related) and IL-6/RORγ/IL-17A (T helper 17 cells-related) in spleen of ApoE^-/-^ mice were detected by western blotting. **(B, C, D, E, F** and **G)** The quantitative comparison of TGF-β1, Smad2/3, Foxp3, IL-6, RORγ and IL-17A between drug treatment groups and model group (n=3). Data were showed as mean ± standard deviation and compared by one-way analysis of variance and following with Fisher’s Least Significant Difference test for individual comparisons. ^n.s.^p > 0.05; ^★^p < 0.05.

To determine whether the QSYQ-dependent Foxp3^+^ Tregs in atherosclerotic plaque came from spleen, we examined the expression of Treg-regulating proteins in the spleen. We failed to observe differences in the expression of TGF-β1 by QSYQ (*P* > 0.05). Surprisingly, however, the expression of mothers against decapentaplegic homolog 2/3 (Smad2/3) and Foxp3 were reduced by high dose QSYQ treatment (*P* < 0.05) (Figure 4B-4D). We suspected that QSYQ treatment might promote Foxp3^+^ Treg cells migration from spleen into aortic plaques and thereby increased the proportion of natural Treg cells in atherosclerotic plaque.

We next examined whether the effect of QSYQ on IL-17A was due to systemic regulation or restricted to the diseased artery. For this purpose, we analyzed the Th17-regulating proteins in the spleen. We found significant reduction of IL-6 in the spleen by the high dose of QSYQ (*P* < 0.05). A similar result was observed for the intracellular key transcription factor RORγ that controls differentiation of Th17 cells (*P* < 0.05). Finally, we found that QSYQ significantly down-regulated the expression of spleen IL-17A (*P* < 0.05) (Figure 4E-4G). These results indicated that QSYQ treatment inhibited Th17-mediated local and systemic inflammatory responses.

## DISCUSSION

The present study showed that QSYQ attenuates atherosclerosis in ApoE^-/-^ mice. The reduction of atherosclerotic lesion formation by QSYQ was associated with promotion of anti-inflammatory Foxp3^+^ Treg cells and inhibition of pro-inflammatory Th17 in atherosclerotic plaque, as well as acceleration of liver cholesterol metabolism. This is the first report of possible immunoregulatory mechanism of QSYQ in a model of atherosclerosis. It provides potential molecular mechanisms for the clinical efficacy of QSYQ in cardiovascular disease in humans afflicted with angina pectoris and myocardial infarction.

QSYQ is a patented drug that has been used in China for the prevention and treatment of coronary heart diseases in the elderly population [17, 24, 25]. Its potential cardioprotective effects were found to be multi-pronged, including attenuating myocardial fibrosis and ameliorating multiple mitochondrial dysfunctions [24, 26–28]. However, the underlying pathology of cardiovascular diseases in most cases is caused by atherosclerosis. Accumulating evidence suggests that innate and adaptive immune responses are involved in every phase of atherosclerosis [2]. In particular, T cells, such as Th1, Th2 and Th17, participate in adaptive immune response to promote the development of atherosclerosis, and to trigger unstable atherosclerotic plaque that are prone to plaque rupture, which induces thrombus formation and myocardial infarction [29]. The major function of Treg cells is to suppress T cell immune responses [30]. Whether QSYQ treatment can affect T cells-mediated immunosuppression and blood lipid metabolism is not clear.

A large number of clinical studies showed that peripheral blood Treg cell subsets in patients with acute coronary syndrome decreased significantly compared with patients with stable angina and associated with reduction in mRNA expression of Foxp3 and TGF-β1 in plasma [31]. In the human atherosclerotic plaque, Foxp3^+^ Treg cells presented all stages of atherosclerosis [32, 33]. Although it has not been clarified whether Treg cells contribute directly or indirectly to the development of atherosclerosis, several studies have been reported that increased number of Treg cells played an athero-protective role in ApoE^-/-^ or LDLR^-/-^ mice [34, 35]. One of the major components of QSYQ, astragalus membranaceus, had anti-asthmatic properties by increasing the population of CD4^+^CD25^+^Foxp3^+^ Treg cells and the mRNA expression of Foxp3 in the lung [36]. We demonstrated here that QSYQ increased the expression of Foxp3 in atherosclerotic plaques and decreased the expression of Smad2/3 and Foxp3 in spleen. We speculated that QSYQ mobilizes Foxp3^+^ Treg cells from the spleen and trigger migration into atherosclerotic plaques and thereby exerts its atheroprotective effects. In addition, there was an increasing trend in the expression of TGF-β1 by two dose of QSYQ in atherosclerotic aorta, but no statistical difference compared with model group. The reason for this result might be due to the few number of sample in each group, or secretion by many other cells but not only Treg cells.

Atherosclerosis and the associated increase in serum IL-17 was accompanied with large numbers of Th17 cells in secondary lymphoid organs [23]. In addition, the expression of IL-17A and RORγt in the diseased artery was significantly up-regulated [37]. Treatment with IL-17A promotes monocyte adhesion to the endothelium in ApoE^-/-^ mice [38]. QSYQ treatment reduced the expression of IL17A in the atherosclerotic aorta, as well as the expression of IL-6/RORγ/IL17A in spleen, which suggested both a local and a systemic anti-inflammatory action of QSYQ.

In addition to the immune imbalance in atherosclerosis, metabolic disorders of blood lipids are key features of atherogenesis. The pathway of LDLR-LXR-α-ABCG5 in liver participates in the transfer of blood cholesterol to bile, and thereby decreased the level of blood VLDL-C and LDL-C [14]. We observed that QSYQ decreased the ratio of liver weight and body weight, and increased the expression of LXR-α and ABCG5. The underlying implication for the effect might be involved in reducing lipid storage in liver and accelerating liver cholesterol excretion.

**Figure 5:**
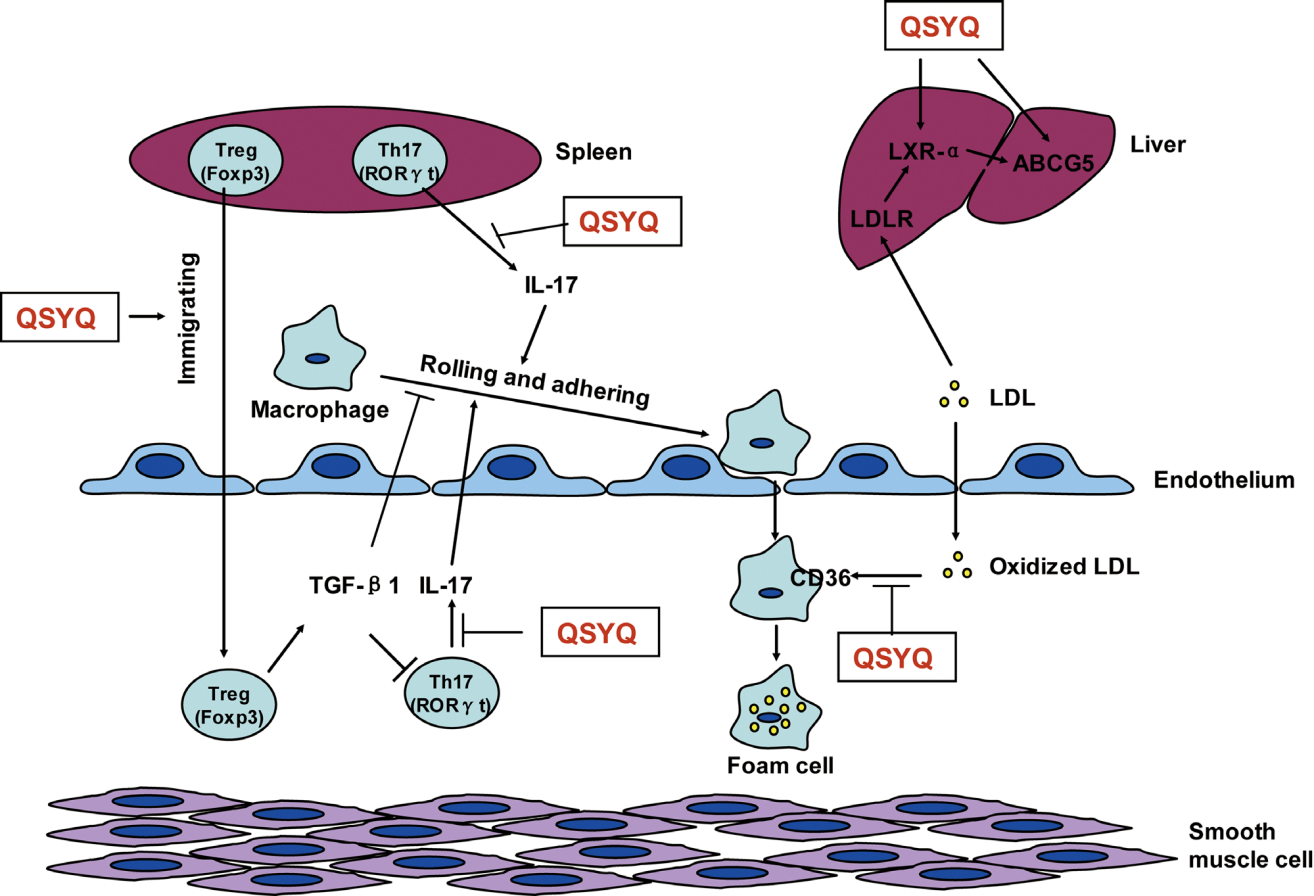
Proposed mechanism underlying QSYQ against atherosclerosis During the development of atherosclerosis, macrophages roll and adhere on the injured endothelium and then infiltrate into sub-endothelium. Macrophages phagocytize oxidized LDL-C and turn into foam cell, which deposit in the sub-endothelium and form the lipid core of atherosclerotic plaque. Treg (Foxp3^+^) and Th17 (RORγt^+^) in spleen immigrate into plaque area. IL-17, the major pro-inflammatory cytokine secreted by Th17 in spleen and atherosclerotic plaque, promotes the rolling and adhering of macrophage, which can be inhibited by TGF-β1. In addition, blood LDL is combined by LDLR of hepatocyte and excreted into bile by LXR-α and ABCG5. The mechanism of QSYQ inhibiting atherosclerosis include promoting Treg immigration into atherosclerotic plaque, inhibiting the secretion of IL17 by Th17 in spleen and plaque, blocking oxidized LDL phagocytized by macrophage through CD36, and increasing the expression of LXR-α and ABCG5 in liver. Notes: QSYQ means QiShenYiQi pill, Treg means regulatory T cell, Th17 means T helper 17 cell, Foxp3 means forkhead box P3, RORγt means RAR-related orphan receptor gamma t, IL-17 means interleukin-17, TGF-β1 means transforming growth factor beta 1, CD36 means cluster of differentiation 36, LDL means low-density lipoproteins, LDLR means LDL receptor, LXR-α means liver X receptor alpha, ABCG5 means ATP-binding cassette sub-family G member 5, Black arrow means promotion or movement, T-type line means inhibition.

According to the above results, the multi-target of QSYQ covered two major event of atherogenesis, that is, modulation of Tregs/Th17-mediated immune responses and promotion of blood lipids metabolism, which might have beneficial effects on reducing or regressing atherosclerosis. The mechanism of QSYQ inhibiting atherosclerosis included promoting Treg immigration into atherosclerotic plaque, inhibiting the secretion of IL17 by Th17 in spleen and plaque, blocking oxidized LDL phagocytized by macrophage through CD36, and increasing the expression of LXR-α and ABCG5 in liver (Figure 5). However, further studies are needed to identify which target or pathway is the top contributor for QSYQ against atherosclerosis.

## MATERIALS AND METHODS

### Animals

Healthy male specific pathogen free (SPF) grade ApoE^-/-^ mice (on the C57BL/6J background), 7 weeks old, weighing 21 to 23g, were purchased from Beijing Huafukang Bioscience Co., LTD (Beijing, China) (Certificate no. SCXK (Jing) 2014-0004). Mice were housed in an air-conditioned atmosphere at 22°C and 50% relative humidity under a 12h dark-light cycle, and received standard diet and water ad libitum. The animal experimental protocol was approved by the ethics committee for administration of experimental animals at the Medical College of Xiamen University (MC20150517). All animal procedures were performed in accordance with the National Institute of Health “Guide for the Care and Use of Laboratory Animals” [39].

### Drug

QiShenYiQi pill (Cat. No.: 140706) was purchased from Tasly Pharmaceutical Co. Ltd. (Tianjin, China), which was produced according to the guidelines of Good Manufacturing Practice and Good Laboratory Practice, and its major components were reported in a previous study [15].

### Experimental design

ApoE^-/-^ mice were divided into three groups: the Model group, the QSYQ low dose group and the QSYQ high dose group (6 mice in each group). All mice were fed a Western-type diet (containing 21% fat and 0.15% cholesterol, produced by Beijing Botaihongda Biotech, Beijing, China) for eight weeks. Mice in drug treatment groups were intraperitoneal injected with QSYQ low dose (0.3 g/kg d, dissolved in 0.3 ml normal saline) and high dose (1.5 g/kg d, dissolved in 0.3 ml normal saline) from the first day of Western diet respectively. Mice in the Model group were intraperitoneal injected with 0.3 ml normal saline. After eight weeks treatment, mice were anesthetized by injecting with 10% chloral hydrate intraperitoneally. The auriculadextra were cut out, then mice were trans-cardiac perfused with 20 ml 1×PBS and followed 20 ml 4% PFA. The aorta, heart, spleen and liver were collected for pathological or molecular biological analysis.

### Blood lipids detection

Blood samples were collected from abdominal aorta of mice at the end of the experiment, then centrifuged for 15 min at the rate of 3000 rpm. Supernatants were taken into pipe bombs and stored at −80°C refrigerator. Serum levels of TC were detected by the COD-PAP method (Cat.no.: A111-1, Nanjing Jiancheng Bioengineering Institute, Nanjing, China). LDL-C were detected by established methods (Cat.no.: A113-1, Nanjing Jiancheng Bioengineering Institute, Nanjing, China). The colorimetric analysis was performed by using a microplate reader (Type: infinite M200 PRO, TECAN, Männedorf, Switzerland).

### Pathological morphological analysis

To evaluate atherosclerotic lesion size, the aortic root was embedded in Tissue Tek (Sakura Finetek USA, Inc., Torrance, CA, USA) and stored at −80°C. Ten micrometer cross frozen sections were prepared, every 10^th^ section was stained with 0.5% Sudan IV (Shenyang Shisan Biotech, Shenyang, China) and used to quantify atherosclerosis. Sections with atherosclerotic lesion were photographical recorded using Image Acquisition System (Type: cellSens Standard 1.9, OLYPUS Corporation, Tokyo, Japan). The plaque area was assessed and calculated by using Image J software (National Institutes of Health, Bethesda, Maryland, USA).

### Western blot

Protein extracts were isolated from tissues in radio-immunoprecipitation assay buffer and protein concentrations were measured using the bicinchoninic acid (BCA) method. Protein supernatants (80 μl) were separated on 10% SDS-polyacrylamide gel (SDS-PAGE) and transferred to nitrocellulose membranes. Target proteins were detected using the anti-target protein antibodies (Table 1) and followed by the secondary antibodies. The enhanced chemiluminescence (ECL) imaging method was used to facilitate the detection of protein bands. Quantification was performed using Quantity One software (Bio-Rad Laboratories, Hercules, California, USA) and the data were showed as the ratio of target protein/β-actin.

**Table 1:** List of antibodies for Western blot

Antibody	Company	Catolog number	Application
Anti-CD36 antibody (EPR 6573)	Abcam	ab133625	1:2000 (WB)
Anti-Foxp3 antibody	Abcam	ab54501	1:2000 (WB)
Anti-TGF beta 1 antibody	GeneTex	GTX110630	1:1000 (WB)
Anti-Smad2/3 antibody (FL425)	Santa Cruz	sc-8332	1:500 (WB)
Anti-IL6 antibody	CST	12912T	1:1000 (WB)
Anti-ROR gamma antibody	Santa Cruz	sc-28559	1:500 (WB)
Anti-IL-17A antibody	R&D systems	AF-421-NA	1:1000 (WB)
Anti-LDLR antibody (EP1553Y)	Abcam	ab52818	1:2000 (WB)
Anti-LXR-α/β antibody (H-144)	Santa Cruz	sc-13068	1:500 (WB)
Anti-ABCG5 antibody (H-300)	Santa Cruz	sc-25796	1:500 (WB)
Anti-β-actin antibody (C4)	Santa Cruz	sc-47778	1:5000 (WB)

### Statistical analysis

All data were normally distributed and were expressed as means ± standard deviation. Groups were compared by one-way analysis of variance (ANOVA) using the Statistical Package for the Social Sciences (SPSS) version 11.5 (SPSS Inc., Chicago, IL, USA). Fisher’s test of least significant difference was used for post-hoc individual comparisons. *P* < 0.05 or 0.01 (two-sided) was considered to be statistically significant.
